# ChromInst: A single cell sequencing technique to accomplish pre-implantation comprehensive chromosomal screening overnight

**DOI:** 10.1371/journal.pone.0251971

**Published:** 2021-05-20

**Authors:** Fang-Fang Gao, Li Chen, Shi-Ping Bo, Ya-Xin Yao, Zhong-Li Xu, Qing-Yu Ding, Peng Zhang, Si-Jia Lu, Jun Ren

**Affiliations:** 1 Department of Research and Development, Yikon Genomics (Suzhou) Company Limited, Suzhou, Jiangsu, PR China; 2 Department of Reproductive Medicine, Affiliated Jinling Hospital, Medicine School of Nanjing University, Nanjing, PR China; University of Massachusetts Amherst, UNITED STATES

## Abstract

Next Generation Sequencing (NGS) is a powerful tool getting into the field of clinical examination. Its preliminary application in pre-implantation comprehensive chromosomal screening (PCCS) of assisted reproduction (test-tube baby) has shown encouraging outcomes that improves the success rate of *in vitro* fertilization. However, the conventional NGS library construction is time consuming. In addition with the whole genome amplification (WGA) procedure in prior, makes the single cell NGS assay hardly be accomplished within an adequately short turnover time in supporting fresh embryo implantation. In this work, we established a concise single cell sequencing protocol, ChromInst, in which the single cell WGA and NGS library construction were integrated into a two-step PCR procedure of ~ 2.5hours reaction time. We then validated the feasibility of ChromInst for overnight PCCS assay by examining 14 voluntary donated embryo biopsy samples in a single sequencing run of Miseq with merely 13M reads production. The good compatibility of ChromInst with the restriction of Illumina sequencing technique along with the good library yield uniformity resulted superior data usage efficiency and reads distribution evenness that ensures precisely distinguish of 6 normal embryos from 8 abnormal one with variable chromosomal aneuploidy. The superior succinctness and effectiveness of this protocol permits its utilization in other time limited single cell NGS applications.

## Introduction

With wide application of PCR, FISH and DNA chip technology, medical examination has moved into the age of molecular diagnosis in the past decade. Recently, high throughput sequencing methods had shown very much promising adding in this age. The constantly throughput increasing of Next Generation Sequencing (NGS) keeps lowering the unit cost of data generation that strongly promotes the application of NGS from an academic research tool toward a powerful clinical examination method. One of such pioneer applications of NGS in clinical setting is single cell sequencing based pre-implantation comprehensive chromosome screen (PCCS) during *in vitro* fertilization (IVF) procedure [1–7].

World widely, the human infertility rate had increased up to 10–15% [8–10]. The assisted reproduction, namely test-tube baby technology, is often the final resolution to against infertility in many cases. However, the success rate of conventional IVF procedure is merely 30–35% in average, and could be even lower to 5–8% in females over age 40 [11–16]. Scientific researches has revealed that embryo chromosomal abnormality is one of major causes of IVF failure [17–20]. It’s estimated that chromosomal abnormalities exist in 40–60% embryos obtained by IVF, and this ratio is increasing with the age of the female [21–28]. Under this circumstance, it’s substantially helpful for IVF success that performs preimplantation genetic testing for aneuploidy (PGT-A) in a PCCS manner, of which a few or even a single cell biopsy from *in vitro* cultured embryos are examined for genome wide chromosomal aneuploidy, then choses the very embryo without chromosomal abnormalities to implant into the mother’s womb [20, 25, 29–31].

A single cell genome counts for a few picogram DNA only. To obtain sufficient initiative materials for NGS library construction, the single cell genome has to be amplified (whole genome amplification, WGA) to millions fold first. The conventional NGS library construction is a time-consuming process including multiple steps of DNA fragmentation, blunt end generation, adapter ligation, PCR amplification etc. The additional WGA process prior to these steps makes single cell sequencing an even more time-consuming process. On the other hand, there is clinical necessity that takes embryo biopsy on the day 3 (or 5) post *in vitro* fertilization then implant a high-quality embryo into the mother’s womb on day 4 (or 6). This requires the PGT-A procedure to be accomplished overnight. It’s almost impossible to achieve this requirement by the conventional single cell sequencing procedure described above.

**Fig 1:**
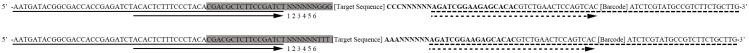
The format of Illumina sequencing library (containing 2 variants) constructed by ChromInst. Sequence of pre-amplification primers is gray shaded. The reverse-complimentary sequence of pre-amplification primer is shown in Bold. The sequences of exponential amplification primer 1 is underlined. The reverse-complimentary sequences of exponential amplification primer 2 is dash-underlined. The sequencing primer (Read 1 primer of Illumina) is indicated by the black arrow. The barcode is a sequence of hexamer nucleotides that is unique for each sample in a given sequencing run. The barcode sequencing primer is indicated by the dashed black arrow. The number 1 to 6 indicate the first 6 base position of a sequencing read, of which the signal emission is used for cluster recognition to initiate a sequencing run (N: either base of A, G, C, or T).

A commercial product (Takara) is able to accomplish single cell WGA simultaneously with the sequencing library construction. The integrated WGA-NGS library construction protocol significantly reduced the time consuming of single cell sequencing thus enable the potential of overnight PGT-A. However, in the Takara’s sequencing library, artificial sequences containing only two types of nucleotide, G and T, were added to flank each DNA fragment to be sequenced. In an Illumina sequencing reaction, the sequencing primer has to extend and read through these artificial bases first then reach to target sequence (Fig 1). Determined by Illumina’s sequencing principle, the signal emission from the first a few base positions of a sequencing read is to be used for cluster recognition to initiate a sequencing run, and the four nucleotide types (A, G, C and T) is preferred to be equally distributed in each of these base positions by default [32]. Therefore, if Takara’s WGA-NGS library product is loaded alone on an Illumina sequencer, as lacking of C and A in the first a few sequencing positions, the cluster recognition will not be accomplished properly thus the sequencing run will end up with failure. The way to solve this problem is to add artificial random fragments, i.e. Phix (Illumina, San Diego, USA), into the sequencing library. In this manner, the library is sequenced in combined with the added in Phix fragments which provides randomness of the four nucleotides thus enables proper cluster recognition. The minimal amount of Phix fragments added in should not be less than 10% of total reads number [33], whereas these reads are certainly not to contribute to effective data.

An overnight PGT-A assay is typically a sequencing run required by one particular patient with embryo biopsy samples up to 15. It’s estimated that minimally 500–1000 K effective reads would be needed to confidently reporting aneuploidy for any given chromosome in PGT-A (internal data, unpublished). Therefore, a sequencer with moderate throughput, such as Illumina Miseq (maximal 15–25 M reads production depend on sequencing reagent version) is a reasonable choice for this purpose. To ensure each biopsy sample being covered by sufficient effective reads is essential for a successful PGT-A assay. Wasting reads on Phix fragments is unwise, that even could be risky when a low version sequencing reagent is used, especially once the reads generation is far less than the maximal potential in a practical sequencing run.

Taken above together, we are aiming to simplify single cell sequencing procedure in fulfill overnight PGT-A requirement of IVF clinic and to ensure maximal data utilization efficiency of NGS sequencing. We established an integrated single-cell WGA-NGS library construction protocol named ‘ChromInst’. As sufficient base randomness is designed in the first a few positions of every sequencing read, the NGS library constructed in this manner is able to be loaded alone in an Illumina sequencer, i.e. Miseq, without adding in Phix fragments. The feasibility to perform overnight PCCS by ChromInst was then demonstrated with a mimic setup of clinical examination.

## Materials and methods

The strategy of single cell WGA in this work was adapted from Multiple Annealing and Looping Based Amplification Cycles (MALBAC) method [34], while the chemical components of cell lysis, pre-amplification, exponential amplification and thermal cycling program remain the same. The pre-amplification primers were modified (Table 1) to be compatible with the adaptor sequences of Illumina sequencing library (Illumina, San Diego, USA, Table 1). Briefly, a single cell was lysed in a 5 μl reaction with 12.5 μg/ml proteinase K, 30mM Tris-HCl (pH7.8), 0.2% Triton X-100, 20 mM KCl and 2 mM EDTA for 15 min. Subsequently, a 30 μl of pre-amplification mixture containing pre-amplification primers and DNA Polymerase was added to the reaction. After the 12 cycles of pre-amplification program (95°C-2min, 95°C-15s, 15°C-50s, 25°C-40s, 35°C-30s, 65°C-40s and 75°C-40s), another 30 μl of exponential amplification mix containing exponential amplification primers was added and subjected for 17 cycles of exponential amplification (94°C-30s, 94°C-20s, 63°C-30s and 72°C-40s). The resulting WGA product with the format of Illumina NGS sequencing library (Fig 1) was then subject to be sequenced on Illumina platform.

**Table 1:** The primer designs.

Designs	Sequences(5’-3’)
Original MALBAC	GTGAGTGATGGTTGAGGTAGTGTGGAGNNNNNGGG
	GTGAGTGATGGTTGAGGTAGTGTGGAGNNNNNTTT
1	*GCTCTTCCGATCT*NNNNNGGG
	*GCTCTTCCGATCT*NNNNNTTT
2	*GCTCTTCCGATCT*NNNNNNGGG *GCTCTTCCGATCT*NNNNNNTTT
3	*GCTCTTCCGATCT*NNNNNNNGGG *GCTCTTCCGATCT*NNNNNNNTTT
4	*GCTCTTCCGATCT*NNNNNNNNGGG *GCTCTTCCGATCT*NNNNNNNNTTT
5	*GCTCTTCCGATCT*NNNNNNNNNGGG *GCTCTTCCGATCT*NNNNNNNNNTTT
exponential amplification primer- modified Illumina adapter sequence	Upstream: AATGATACGGCGACCACCGAGATCTACACTCTTTCCCTACACGAC*GCTCTTCCGATCT*
	Downstream: CAAGCAGAAGACGGCATACGAGAT[Barcode]GTGACTGGAGTTCAGACGTGT*GCTCTTCCGATCT*

The 27 bp constant sequence of original MALBAC pre-amplification primer is underlined. The 13 bp constant sequence of the 3’ end sequence of Illumina exponential amplification upstream primer used to replace the 27 bp constant sequence of original MALBAC are shown in Italic. In searching optimal primer length for effective sequencing library construction, random nucleotides in variable length were inserted into the pre-amplification primer (design 1–5, Table 1). Barcode is a sequence of hexamer nucleotide unique for each sample in a given sequencing run. “N” represents either base A, G, C, or T.

The works in this study were divided into three phases. In the first phase, 50 picograms human genomic DNA were used as biological sample in protocol optimization. Conventional agarose gel (2%) electrophoresis were used to evaluate the efficiency and size range of WGA product (namely, sequencing library).

In the second phase, single cells of *in vitro* cultured human lymphocytes (GM12878) were picked by mouth pipetting under stereo microscope, then used as biological samples. Illumina NGS sequencer MiSeq was used to evaluate the quality of sequencing library upon the integrated WAG-NGS library construction process. To apply multiple samples in a single sequencing run, unique barcode sequences were incorporated respectively into the primers of exponential amplification (of WGA) for each sample. After the WGA-NGS library construction, the libraries of each sample were mixed at equal volume and then purified with MagBead DNA Purification Kit (CoWin Biotech, Beijing, China) followed the manufacture’s protocol. The DNA concentration of purified library mixture was determined by Qubit^®^2.0 (Thermo Fisher Scientific, Waltham, MA, USA) followed the manufacture’s protocol. To determine the amount of library to be loaded into the sequencer, the molarity of the library was calculated from the Qubit results (in nanogram per microliter). The library was sequenced alone without adding of artificial random fragments (Phix). The MiSeq Reagent Kit v3 (theoretical Maximal 25 M reads production) was used and SE75 sequencing program was applied at early ending of 55bp following Illumina’s sequencing protocol.

For data analysis, the sequencing raw data (in.bcl format) was demultiplexed and converted to the FASTQ format using a Perl script configure BclToFastq.pl in CASAVA(Consensus Assessment of Sequence and Variation, version 1.8.2) [35] package based on the sample sheet information. Illumina adaptors, low quality bases (bases with quality score less than 20) were removed from the FASTQ file using Trimmomatic [36] (version 0.35). High quality reads were mapped to human reference genome (hg19) [37] using BWA (Burrows-Wheeler Alignment Tool, version 0.7.12-r1039) with default parameters [38]. The mapped reads were sorted and converted to binary format.bam using SamtoBam.jar in Picard (version1.119) package [38].

In the third phase, trophectoderm biopsy samples of *in vitro* cultured human embryo donated from an anonymous volunteer were examined to validate the application of the protocol established in clinical setting of overnight PCCS. The human embryo biopsies were collected on May 5, 2017 and testing and analysis were carried out from that day to May 6. We do not have access to information that can identify individual participants during or after data collection. This study was approved by the Ethics Committee of Nanjing Jinling Hospital (reference number 2016NZKY-028-02), and written informed consent was obtained prior to embryo analysis. Biopsy sample duplicates from each embryo were examined separately by the protocol established above and original MALBAC WGA followed by routine library construction based NGS descripted previously [7, 34, 39]. To validate whether the library yield uniformity enables sufficient reads coverage among multiple samples over limited total reads production, MiSeq reagent kit v2 with a theoretical maximal output of 15M reads, was used in the test. In addition to the resulting data observed in the phase two for library quality evaluation, the chromosomal copy number variation (CNV) of each embryo biopsy was analyzed by the program descripted in previous publication [35–38, 40]. Briefly, unique mapped reads were extracted from the alignment reads (.bam file) using Samtools (version 1.2.1) [41]. The whole reference genome was divided into a serial of 200Kb or 1M bins. Reads number, GC content were calculated within each bin. GC bias correction was processed for every 1% GC content by local Perl [42] scripts. The R (version 3.0.0) [43] was used to generate the graphs of the GC corrected relative reads number (RRN) of each bin to visualize copy number variations. The CNV identified was subsequently compared between the protocol established in this work and routine MALBAC-NGS based PGT-A.

## Results

**Fig 2:**
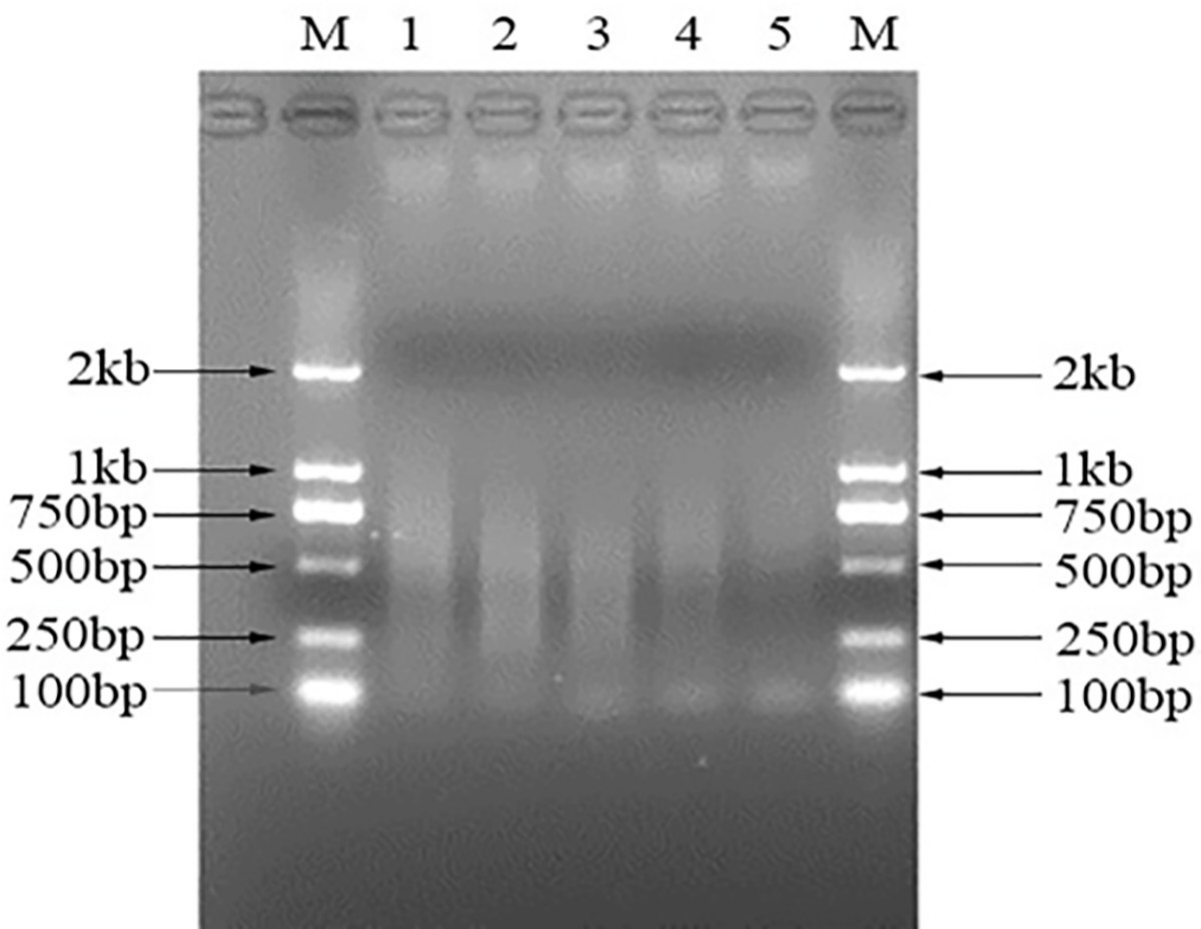
WGA-NGS library construction result from variable pre-amplification primer designs. Fifty picograns human genomic DNA was used as amplification template. The amplification products are visualized on a 2% agarose gel as routine. M: DM2000 DNA Marker; Lane 1–5: the WGA products of design 1–5.

In order to combine the MALBAC WGA procedure and Illumina sequencing library construction into an integrated process, the 5’ end 27 bp of MALBAC pre-amplification primer sequence (Table 1 underlined sequence) was firstly replaced by the 3’ end sequence of the Illumina adapter. To determine the optimal sequence length should be used for random nucleotide of pre-amplification primer, length serials of 5bp, 6bp, 7bp, 8bp and 9bp (Table 1, design 1, 2, 3, 4 and 5) were tested. The experimental results showed that, while the design 1 produced amplification product in wider size range and the design 4 and 5 generated lower yield, the design 2 and 3 produces shorter amplification products at higher yield (Fig 2). Thus, the design 2 was chosen for the subsequent protocol development.

In the phase two of the study, single cells were picked from *in vitro* cultured human lymphocytes then the WGA protocol optimized above was applied to each of the cell. Fifteen single cells were used in the test. To minimize examination turnover time, we simply pooled the library from each sample at equal volume instead of the conventional pooling strategy that performs qPCR quantification to each library then pools them in equal molar manner. The pooled library was loaded on MiSeq sequencer without adding artificial random fragments (Phix). The sequencing run was successfully completed that 26M reads at Q30>90% were yield with average length of 55bp. The average reads yield of 15 samples was 1.6M, with maximal reads of 2.3M and minim reads of 1.3M (Table 2). The data that support the findings of this study have been deposited into CNGB Sequence Archive (CNSA) [44] of China National GeneBank DataBase (CNGBdb) with accession number CNP0001733. The evenness of sequencing reads distribution over the samples (less than two folds variation) demonstrated the robustness of the protocol on library yield uniformity. With the unique mapping rate more than 80% (Table 2), the library yield uniformity ensures each single cell sample is covering by sufficient data. At this point, a single cell NGS sequencing protocol was established which was potentially able to accomplish overnight PCCS. We called it ‘ChromInst’ to emphasize its capability to perform chromosomal screening in a swift manner.

**Table 2:** QC data of 15 single cells in a single run of Miseq sequencing.

sample	raw reads	GC%	high quality of raw	Mapping rate	Mapped of raw	Unique mapped of raw
**1**	1,514,213	40	96.04%	94.86%	91.1%	84.03%
**2**	1,616,350	40	95.79%	94.09%	90.14%	83.02%
**3**	1,837,612	40	96.07%	94.65%	90.93%	83.83%
**4**	1,495,434	40	96.12%	93.71%	90.07%	82.98%
**5**	2,381,030	40	96.32%	95%	91.51%	84.36%
**6**	1,944,712	40	96.28%	94.85%	91.32%	84.22%
**7**	1,992,210	40	96.27%	93.99%	90.48%	83.46%
**8**	2,174,977	40	96.7%	95.64%	92.48%	85.49%
**9**	1,700,875	40	96.46%	94.65%	91.31%	84.43%
**10**	1,567,411	40	96.06%	94.62%	90.9%	83.99%
**11**	1,505,962	40	96.18%	94.24%	90.64%	83.83%
**12**	2,110,626	40	96.24%	94.85%	91.28%	84.3%
**13**	1,481,780	41	96.09%	89.85%	86.34%	79.84%
**14**	1,888,895	40	96.14%	93.62%	90.01%	83.1%
**15**	1,389,200	40	95.88%	94.47%	90.58%	83.71%
**CV**^**a**^	16.9%	0.6%	0.2%	1.4%	1.5%	1.5%

^a^CV: coefficient of variation

To validate whether the ChromInst protocol is indeed able to achieve overnight PCCS as clinically needed, 14 embryo biopsy samples donated by an anonymous volunteer were tested in a single MiSeq run. Based on our previous PGT-A application, a minimal read of 500K is adequate for comprehensive chromosomal aneuploidy screening (unpublished data). As the phase two results showed satisfied library uniformity among samples, we decided to perform the phase three test in a low read yield manner by using MiSeq reagent kit v2 that gives maximal reads production at 15M only, in examining the practicability of the protocol.

While the embryo biopsy samples were arrived in our laboratory at 6 pm, the WGA-NGS library construction was accomplished at 9:30 pm. After equal volume pooling, purification and quantification, the library mixture of 14 samples (in addition with positive and negative controls) was loaded onto the MiSeq sequencer at 11:30 pm. The sequencing run was accomplished at 5:30 AM on the next day and data analysis was accomplished at 6:00 AM. The timeline of the whole procedure and major measures to reduce time cost is summarized in the Table 3.

**Table 3:** The timeline of overnight PGT-A procedure in this work.

Steps	Duration (hr)	Hands on time (hr)
Reagent preparation upon sample receiving	0.5	0.5
WGA-NGS library construction	3	0.5
Library pooling, purification, quantification and loading into sequencer	2	2
Sequencing running	6	0
Data analysis	0.5	0.5
Total	12	3.5
Summary of major time saving measures	Integrate WGA and NGS library construction to a two-step PCR process of ~2.5 hr reaction time	
	Library pooling at equal volume of each sample instead of conventional qPCR quantification	
	A short length single-ended sequencing run of 55 bp	

**Fig 3:**
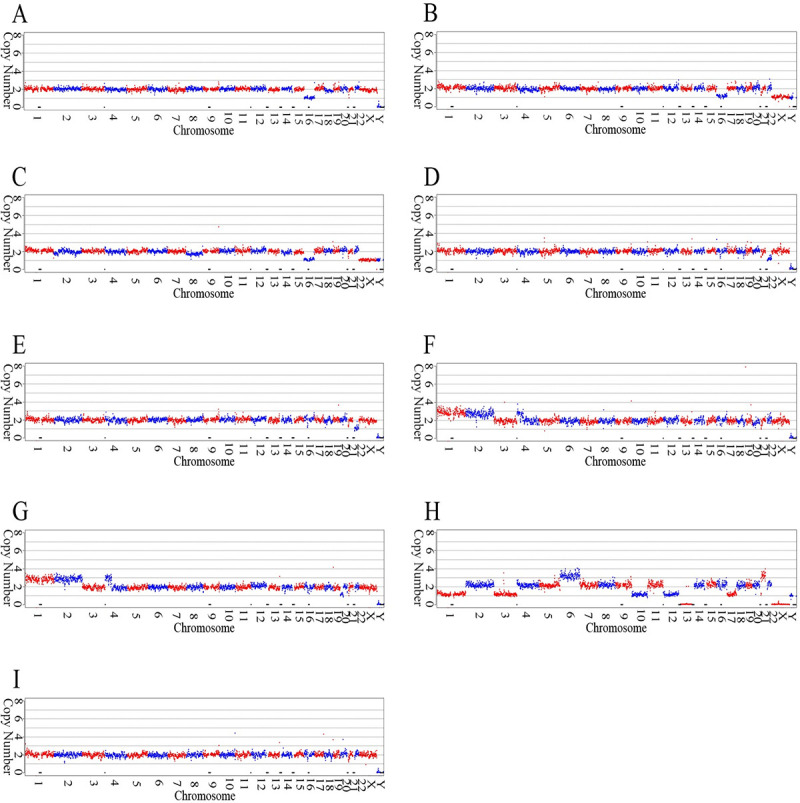
Chromosomal CNV examination of embryo biopsy samples. Chromosomes are aligned along X axis from 1 to 22 with X and Y at the end. Chromosomal copy number was set as Y axis. Aneuploidy was identified from 8 of the 14 embryos examined, which are as shown in the panel A: Sample 1; B: Sample 4; C: Sample 5; D: Sample 6; E: Sample 7; F: Sample 8; G: Sample 9; H: Sample13. Panel I shows a random example of normal diploid genome (Sample 11).

The sequencing run was successfully accomplished with 13M reads at Q30 > 90% and an average read length of 55 bp. The average reads yield per samples was 871K, with maximal reads of 1.42M and minim reads of 541K (Table 4). Chromosomal CNVs were identified in 8 samples of the 14 embryos examined, while the other 6 samples were euploidy genome (Fig 3). No significant differences were observed in the paired comparison between the results above and the routine MALBAC-NGS based assay (data no shown).

**Table 4:** QC data of 14 embryo biopsy sequencing.

sample	Raw reads	High quality GC%	High quality of raw	Mapping rate	Mapped of raw	Unique mapped reads	Unique mapped of raw
**1**	765,904	41	97.11%	97.17%	94.36%	621,999	81.21%
**2**	569,266	41	97.35%	97.43%	94.85%	457,252	80.32%
**3**	1,151,433	42	97.62%	97.87%	95.53%	954,220	82.87%
**4**	848,321	41	97.39%	96.82%	94.29%	680,302	80.19%
**5**	1,013,240	41	97.31%	97.32%	94.70%	823,884	81.31%
**6**	724,464	41	97.38%	97.03%	94.48%	585,789	80.86%
**7**	860,599	41	97.28%	97.61%	94.96%	709,675	82.46%
**8**	759,668	42	97.47%	97.05%	94.59%	619,175	81.51%
**9**	818,820	41	97.15%	97.67%	94.88%	676,557	82.63%
**10**	541,819	42	97.59%	97.60%	95.26%	439,336	81.09%
**11**	1,018,913	42	97.45%	97.78%	95.28%	837,823	82.23%
**12**	813,351	41	97.41%	97.25%	94.73%	657,646	80.86%
**13**	1,420,866	41	97.25%	90.75%	88.25%	1,086,243	76.45%
**14**	899,714	42	97.18%	96.31%	93.60%	732,360	81.40%
**NC**^**a**^	33,236	43	90.89%	57.68%	52.43%	15,001	45.13%
**PC**^**b**^	959,591	41	97.62%	95.13%	92.87%	764,751	79.70%
**CV**^**c**^ **(except NC)**	25.1%	1.2%	0.2%	1.8%	1.9%	24.0%	1.9%

^a^NC: negative control, reagent only

^b^PC: positive control, 20 pg genomic DNA

^c^CV: coefficient of variation

## Discussion

In this work, we established a modified MALBAC protocol namely ChromInst, which combines the single-cell WGA and Illumina NGS library construction into an integrated procedure. In addition to time saving, the good compatibility of ChromInst with Illumina sequencing principle along with its good library yield uniformity provided superior data usage efficiency and reads distribution evenness. All these advantages allowed the feasibility of using ChromInst to perform overnight PCCS on multiple embryo biopsies in a sequencing run with relatively low throughput, such as by MiSeq reagent kit v2.

A collective evidence indicate that PGT-A in PCCS manner is beneficial to improve the success rate of IVF [18, 20, 22, 23, 29, 45–47], and NGS is a promising technique to perform PCCS [1, 2, 5, 6, 48, 49]. Nevertheless, the conventional NGS-PGT-A protocol takes days to accomplish the assay. As the optimal time for embryo biopsy is on the day 5 post *in vitro* fertilization and the timing for embryo implantation should be on the day 6 [50], in the conventional NGS based PGT-A, the post biopsy embryos have to be frozen in liquid nitrogen before the examination results turnover. The influence of embryo frozen is remaining in controversial [51–55]. A recent study published in the New England Journal of Medicine demonstrated that frozen embryo resulted higher IVF successful rate [53]. However, the same study also revealed that embryo frozen may cause higher chance of delivering overweight babies and three folds higher chance of gestational pre-eclampsia. Therefore, the benefit and risk balance between frozen and fresh embryo implantation is still to be further evaluated. At this moment, both frozen and fresh embryo implantation are applied in clinical practices. Furthermore, for a patient failed of pregnancy in the first attempt of conventional IVF procedure, her leftover embryos (have to be frozen) are worthy to PGT-A prior to the next try of implantation. Once defrosted, one should avoid the embryos from frozen again before implantation when at all possible. In this circumstance, the overnight PGT-A can be the only way to perform PCCS to improve the chance of IVF success in the second attempt of embryo implantation. As the result, it is necessary to establish an overnight PGT-A procedure that enables fresh embryo implantation in the reality.

The WGA amplification of ChromInst is based on that of MALBAC [34]. MALBAC is a relatively new WGA method which was invented in 2012. It’s amplification uniformity and allele dropout (ADO) rate are superior to previous WGA methods, such as Degenerate Oligonucleotide Primer PCR (DOP-PCR) and Multiple Displacement Amplification (MDA) [56–58] for single cell genome amplification. Two major modifications of the ChromInst protocol were substituting the 27 bp constant part of MALBAC’s pre-amplification primer sequence to the 13 bp (Table 1 Design 2) of the 3’ part of Illumina’s library construction adaptor and were extending the straight 5 “N” to 6 “N” (Table 1, Fig 1). There modifications kept the amplicon looping forming feature of MALBAC in the pre-amplification stage thus inherited the innate high uniformity feature in MALBAC WGA, while accomplishes the sequencing library construction simultaneously. As illustrated in the Fig 3, the WGA uniformity of ChromInst was sufficient for effectively detecting chromosomal aneuploidy from embryonic biopsy by NGS.

When multiple samples are simultaneously examined on a sequencer with limited reads yield capacity, the reads number uniformity over each sample in the sequencing run is important to ensure all samples covered by sufficient sequencing data. Normally, the good uniformity of inter-sample reads yield is achieved by qPCR quantification to the sequencing library of each sample, then pool the samples in equal molar manner. The qPCR is however not generally available in the clinical laboratory setting and the extra time cost is highly undesirable for an overnight assay constrained by time. In our protocol presented, by merit of the reaction constancy of ChromInst, sequencing library of each sample was simply pooled at equal volume prior to load into the sequencer. And the resulted reads yield uniformity was fairly acceptable in term of either reads number on each sample or reads number CV among samples (Tables 2 and 4). Consequently, the ploidy of the 14 tested embryos, including 6 euploidy and 8 aneuploidy samples, were all well identified by merely 13M total reads yield from the sequencing run (Fig 3). The concise experimental protocol and satisfactory examination results demonstrated that the presented approaches are well practical to accomplish PCCS overnight in clinical laboratory setting.

In principle, the sequencing library construction of ChromInst belongs to the category of “random short fragmentation” that is uncapable of detecting the chromosomal structural rearrangement, such as inversion and translocation. In Mate-Pair sequencing, self-cyclization of long DNA fragment is conducted prior to library construction, thus chromosomal structural rearrangement can be identified by sophisticated strategy in subsequent sequencing data analysis [59]. However, the integration of single cell WGA with Mate-Pair library construction remains a great challenge. We also observed that the ADO rate of ChromInst was not as good as in the original MALBAC (unpublished data), presumably due to the suboptimal design of the pre-amplification primers in comparison to the original MALBAC. For this reason, ChromInst may not be suitable in detecting SNPs or point mutations like other single-cell NGS applications.

In summary, a modified MALBAC protocol, ChromInst, was established in this work, which combines single cell WGA and Illumina sequencing library construction into an integrated procedure that reduces the time cost of WGA plus sequencing library construction to an approximately 2.5 hours reaction time. By maintaining the merit of high amplification uniformity of original MALBAC WGA and additional conciseness and superior data efficiency, ChromInst fulfills the clinical necessity of NGS based overnight PCCS. These advantages are also permitting the application of ChromInst in other pressing single-cell NGS applications.

## Supporting information

S1 Raw images The original image of Fig 2. (PDF)

## References

[1] Treff NR, Forman EJ, Scott RT Jr. Next-generation sequencing for preimplantation genetic diagnosis. Fertil Steril. 2013;99(6):e17–8. 10.1016/j.fertnstert.2013.02.034 . 23481279

[2] Wells D. Next-generation sequencing: the dawn of a new era for preimplantation genetic diagnostics. Fertil Steril. 2014;101(5):1250–1. 10.1016/j.fertnstert.2014.03.006 . 24786744

[3] Bono S, Biricik A, Spizzichino L, Nuccitelli A, Minasi MG, Greco E, et al. Validation of a semiconductor next-generation sequencing-based protocol for preimplantation genetic diagnosis of reciprocal translocations. Prenat Diagn. 2015;35(10):938–44. 10.1002/pd.4665 . 26243475

[4] Kung A, Munne S, Bankowski B, Coates A, Wells D. Validation of next-generation sequencing for comprehensive chromosome screening of embryos. Reprod Biomed Online. 2015;31(6):760–9. 10.1016/j.rbmo.2015.09.002 . 26520420

[5] Fiorentino F, Biricik A, Bono S, Spizzichino L, Cotroneo E, Cottone G, et al. Development and validation of a next-generation sequencing-based protocol for 24-chromosome aneuploidy screening of embryos. Fertil Steril. 2014;101(5):1375–82. 10.1016/j.fertnstert.2014.01.051 . 24613537

[6] Fiorentino F, Bono S, Biricik A, Nuccitelli A, Cotroneo E, Cottone G, et al. Application of next-generation sequencing technology for comprehensive aneuploidy screening of blastocysts in clinical preimplantation genetic screening cycles. Hum Reprod. 2014;29(12):2802–13. 10.1093/humrep/deu277 . 25336713

[7] Li N, Wang L, Wang H, Ma M, Wang X, Li Y, et al. The Performance of Whole Genome Amplification Methods and Next-Generation Sequencing for Pre-Implantation Genetic Diagnosis of Chromosomal Abnormalities. J Genet Genomics. 2015;42(4):151–9. 10.1016/j.jgg.2015.03.001 . 25953353

[8] Dunson DB, Baird DD, Colombo B. Increased infertility with age in men and women. Obstet Gynecol. 2004;103(1):51–6. 10.1097/01.AOG.0000100153.24061.45 . 14704244

[9] Mascarenhas MN, Flaxman SR, Boerma T, Vanderpoel S, Stevens GA. National, regional, and global trends in infertility prevalence since 1990: a systematic analysis of 277 health surveys. PLoS Med. 2012;9(12):e1001356. 10.1371/journal.pmed.1001356 23271957 PMC3525527

[10] Balasch J. Ageing and infertility: an overview. Gynecol Endocrinol. 2010;26(12):855–60. 10.3109/09513590.2010.501889 . 20642380

[11] Stern JE, Brown MB, Wantman E, Kalra SK, Luke B. Live birth rates and birth outcomes by diagnosis using linked cycles from the SART CORS database. J Assist Reprod Genet. 2013;30(11):1445–50. 10.1007/s10815-013-0092-0 24014215 PMC3879943

[12] Gunby J, Daya S, Fertility IVFDGotC, Andrology S. Assisted reproductive technologies (ART) in Canada: 2002 results from the Canadian ART Register. Fertil Steril. 2006;86(5):1356–64. 10.1016/j.fertnstert.2006.04.030 . 17070192

[13] Mansour R, Ishihara O, Adamson GD, Dyer S, de Mouzon J, Nygren KG, et al. International Committee for Monitoring Assisted Reproductive Technologies world report: Assisted Reproductive Technology 2006. Hum Reprod. 2014;29(7):1536–51. 10.1093/humrep/deu084 . 24795090

[14] Ishihara O, Adamson GD, Dyer S, de Mouzon J, Nygren KG, Sullivan EA, et al. International committee for monitoring assisted reproductive technologies: world report on assisted reproductive technologies, 2007. Fertil Steril. 2015;103(2):402–13 e11. 10.1016/j.fertnstert.2014.11.004 . 25516078

[15] European IVFMC, European Society of Human R, Embryology, Kupka MS, D’Hooghe T, Ferraretti AP, et al. Assisted reproductive technology in Europe, 2011: results generated from European registers by ESHRE. Hum Reprod. 2016;31(2):233–48. 10.1093/humrep/dev319 . 26740578

[16] Dahdouh EM, Balayla J, Garcia-Velasco JA. Impact of blastocyst biopsy and comprehensive chromosome screening technology on preimplantation genetic screening: a systematic review of randomized controlled trials. Reprod Biomed Online. 2015;30(3):281–9. 10.1016/j.rbmo.2014.11.015 . 25599824

[17] Munne S. Chromosome abnormalities and their relationship to morphology and development of human embryos. Reprod Biomed Online. 2006;12(2):234–53. 10.1016/s1472-6483(10)60866-8 . 16478592

[18] Gianaroli L, Magli MC, Ferraretti AP, Tabanelli C, Trombetta C, Boudjema E. The role of preimplantation diagnosis for aneuploidies. Reprod Biomed Online. 2002;4 Suppl 3:31–6. 10.1016/s1472-6483(12)60113-8 . 12470562

[19] Munne S, Howles CM, Wells D. The role of preimplantation genetic diagnosis in diagnosing embryo aneuploidy. Curr Opin Obstet Gynecol. 2009;21(5):442–9. 10.1097/GCO.0b013e32832fad73 . 19606031

[20] Rubio C, Rodrigo L, Perez-Cano I, Mercader A, Mateu E, Buendia P, et al. FISH screening of aneuploidies in preimplantation embryos to improve IVF outcome. Reprod Biomed Online. 2005;11(4):497–506. 10.1016/s1472-6483(10)61146-7 . 16274616

[21] Munne S, Alikani M, Tomkin G, Grifo J, Cohen J. Embryo morphology, developmental rates, and maternal age are correlated with chromosome abnormalities. Fertil Steril. 1995;64(2):382–91. . 7615118

[22] Schoolcraft WB, Katz-Jaffe MG, Stevens J, Rawlins M, Munne S. Preimplantation aneuploidy testing for infertile patients of advanced maternal age: a randomized prospective trial. Fertil Steril. 2009;92(1):157–62. 10.1016/j.fertnstert.2008.05.029 . 18692827

[23] Milan M, Cobo AC, Rodrigo L, Mateu E, Mercader A, Buendia P, et al. Redefining advanced maternal age as an indication for preimplantation genetic screening. Reprod Biomed Online. 2010;21(5):649–57. 10.1016/j.rbmo.2010.06.020 . 20864410

[24] Harton GL, Munne S, Surrey M, Grifo J, Kaplan B, McCulloh DH, et al. Diminished effect of maternal age on implantation after preimplantation genetic diagnosis with array comparative genomic hybridization. Fertil Steril. 2013;100(6):1695–703. 10.1016/j.fertnstert.2013.07.2002 . 24034939

[25] Hardarson T, Hanson C, Lundin K, Hillensjo T, Nilsson L, Stevic J, et al. Preimplantation genetic screening in women of advanced maternal age caused a decrease in clinical pregnancy rate: a randomized controlled trial. Hum Reprod. 2008;23(12):2806–12. 10.1093/humrep/den217 . 18583331

[26] Munne S, Chen S, Colls P, Garrisi J, Zheng X, Cekleniak N, et al. Maternal age, morphology, development and chromosome abnormalities in over 6000 cleavage-stage embryos. Reprod Biomed Online. 2007;14(5):628–34. 10.1016/s1472-6483(10)61057-7 . 17509208

[27] Magli MC, Gianaroli L, Ferraretti AP. Chromosomal abnormalities in embryos. Mol Cell Endocrinol. 2001;183 Suppl 1:S29–34. 10.1016/s0303-7207(01)00574-3 . 11576729

[28] Kuliev A, Cieslak J, Ilkevitch Y, Verlinsky Y. Chromosomal abnormalities in a series of 6,733 human oocytes in preimplantation diagnosis for age-related aneuploidies. Reprod Biomed Online. 2003;6(1):54–9. 10.1016/s1472-6483(10)62055-x . 12626143

[29] Lee HL, McCulloh DH, Hodes-Wertz B, Adler A, McCaffrey C, Grifo JA. In vitro fertilization with preimplantation genetic screening improves implantation and live birth in women age 40 through 43. J Assist Reprod Genet. 2015;32(3):435–44. 10.1007/s10815-014-0417-7 25578536 PMC4363234

[30] Keltz MD, Vega M, Sirota I, Lederman M, Moshier EL, Gonzales E, et al. Preimplantation genetic screening (PGS) with Comparative genomic hybridization (CGH) following day 3 single cell blastomere biopsy markedly improves IVF outcomes while lowering multiple pregnancies and miscarriages. J Assist Reprod Genet. 2013;30(10):1333–9. 10.1007/s10815-013-0070-6 23949213 PMC3824853

[31] Rubio C, Bellver J, Rodrigo L, Bosch E, Mercader A, Vidal C, et al. Preimplantation genetic screening using fluorescence in situ hybridization in patients with repetitive implantation failure and advanced maternal age: two randomized trials. Fertil Steril. 2013;99(5):1400–7. 10.1016/j.fertnstert.2012.11.041 . 23260857

[32] Using a PhiX Control for HiSeq® Sequencing Runs. Available from: https://support.illumina.com.cn/content/dam/illumina-marketing/documents/products/technotes/hiseq-phix-control-v3-technical-note.pdf.

[33] miseq denature dilute libraries guide 15039740 v10. Available from: https://support.illumina.com.cn/content/dam/illumina-support/documents/documentation/system_documentation/miseq/miseq-denature-dilute-libraries-guide-15039740-10.pdf.

[34] Zong C, Lu S, Chapman AR, Xie XS. Genome-wide detection of single-nucleotide and copy-number variations of a single human cell. Science. 2012;338(6114):1622–6. 10.1126/science.1229164 23258894 PMC3600412

[35] CASAVA. Available from: http://www.illumina.com/.

[36] Bolger AM, Lohse M, Usadel B. Trimmomatic: a flexible trimmer for Illumina sequence data. Bioinformatics. 2014;30(15):2114–20. 10.1093/bioinformatics/btu170 24695404 PMC4103590

[37] UCSC. Available from: http://genome.ucsc.edu/.

[38] Picard Available from: http://picard.sourceforge.net/.

[39] Huang J, Yan L, Fan W, Zhao N, Zhang Y, Tang F, et al. Validation of multiple annealing and looping-based amplification cycle sequencing for 24-chromosome aneuploidy screening of cleavage-stage embryos. Fertil Steril. 2014;102(6):1685–91. 10.1016/j.fertnstert.2014.08.015 . 25241375

[40] Li H, Durbin R. Fast and accurate short read alignment with Burrows-Wheeler transform. Bioinformatics. 2009;25(14):1754–60. Epub 2009/05/20. 10.1093/bioinformatics/btp324 19451168 PMC2705234

[41] Li H, Handsaker B, Wysoker A, Fennell T, Ruan J, Homer N, et al. The Sequence Alignment/Map format and SAMtools. Bioinformatics. 2009;25(16):2078–9. 10.1093/bioinformatics/btp352 19505943 PMC2723002

[42] The Perl Programming Language. Available from: http://www.perl.org/.

[43] The R Project for Statistical Computing. Available from: http://www.r-project.org/.

[44] Guo X, Chen F, Gao F, Li L, Liu K, You L, et al. CNSA: a data repository for archiving omics data. Database: the journal of biological databases and curation. 2020. 10.1093/database/baaa055 32705130 PMC7377928

[45] Kahraman S, Bahce M, Samli H, Imirzalioglu N, Yakisn K, Cengiz G, et al. Healthy births and ongoing pregnancies obtained by preimplantation genetic diagnosis in patients with advanced maternal age and recurrent implantation failure. Hum Reprod. 2000;15(9):2003–7. 10.1093/humrep/15.9.2003 . 10967004

[46] Munne S, Chen S, Fischer J, Colls P, Zheng X, Stevens J, et al. Preimplantation genetic diagnosis reduces pregnancy loss in women aged 35 years and older with a history of recurrent miscarriages. Fertil Steril. 2005;84(2):331–5. 10.1016/j.fertnstert.2005.02.027 . 16084873

[47] Chen M, Wei S, Hu J, Quan S. Can Comprehensive Chromosome Screening Technology Improve IVF/ICSI Outcomes? A Meta-Analysis. PLoS One. 2015;10(10):e0140779. 10.1371/journal.pone.0140779 26470028 PMC4607161

[48] Zheng H, Jin H, Liu L, Liu J, Wang WH. Application of next-generation sequencing for 24-chromosome aneuploidy screening of human preimplantation embryos. Mol Cytogenet. 2015;8:38. 10.1186/s13039-015-0143-6 26085841 PMC4469409

[49] Russo CD, Di Giacomo G, Cignini P, Padula F, Mangiafico L, Mesoraca A, et al. Comparative study of aCGH and Next Generation Sequencing (NGS) for chromosomal microdeletion and microduplication screening. J Prenat Med. 2014;8(3–4):57–69. 26266003 PMC4510565

[50] Brezina PR, Ke RW, Kutteh WH. Preimplantation genetic screening: a practical guide. Clin Med Insights Reprod Health. 2013;7:37–42. 10.4137/CMRH.S10852 24453517 PMC3888082

[51] Pereira N, Petrini AC, Lekovich JP, Schattman GL, Rosenwaks Z. Comparison of perinatal outcomes following fresh and frozen-thawed blastocyst transfer. Int J Gynaecol Obstet. 2016;135(1):96–100. 10.1016/j.ijgo.2016.04.007 . 27388034

[52] Roque M, Valle M, Guimaraes F, Sampaio M, Geber S. Freeze-all policy: fresh vs. frozen-thawed embryo transfer. Fertil Steril. 2015;103(5):1190–3. 10.1016/j.fertnstert.2015.01.045 . 25747130

[53] Chen ZJ, Shi Y, Sun Y, Zhang B, Liang X, Cao Y, et al. Fresh versus Frozen Embryos for Infertility in the Polycystic Ovary Syndrome. N Engl J Med. 2016;375(6):523–33. 10.1056/NEJMoa1513873 . 27509101

[54] Shah MS, Caballes M, Lathi RB, Baker VL, Westphal LM, Milki AA. In vitro fertilization outcomes after fresh and frozen blastocyst transfer in South Asian compared with white women. Fertil Steril. 2016;105(6):1484–7. 10.1016/j.fertnstert.2016.02.027 . 26952781

[55] Ma GC, Chen HF, Yang YS, Lin WH, Tsai FP, Lin CF, et al. A pilot proof-of-principle study to compare fresh and vitrified cycle preimplantation genetic screening by chromosome microarray and next generation sequencing. Mol Cytogenet. 2016;9:25. 10.1186/s13039-016-0238-8 27006692 PMC4802588

[56] Telenius H, Carter NP, Bebb CE, Nordenskjold M, Ponder BA, Tunnacliffe A. Degenerate oligonucleotide-primed PCR: general amplification of target DNA by a single degenerate primer. Genomics. 1992;13(3):718–25. 10.1016/0888-7543(92)90147-k . 1639399

[57] Dean FB, Nelson JR, Giesler TL, Lasken RS. Rapid amplification of plasmid and phage DNA using Phi 29 DNA polymerase and multiply-primed rolling circle amplification. Genome Res. 2001;11(6):1095–9. 10.1101/gr.180501 11381035 PMC311129

[58] Cheung VG, Nelson SF. Whole genome amplification using a degenerate oligonucleotide primer allows hundreds of genotypes to be performed on less than one nanogram of genomic DNA. Proc Natl Acad Sci U S A. 1996;93(25):14676–9. 10.1073/pnas.93.25.14676 8962113 PMC26194

[59] Dong Z, Jiang L, Yang C, Hu H, Wang X, Chen H, et al. A robust approach for blind detection of balanced chromosomal rearrangements with whole-genome low-coverage sequencing. Human mutation. 2014;35(5):625–36. 10.1002/humu.22541 . 24610732

