# Supplementary material for: ChromInst: A single cell sequencing technique to accomplish pre-implantation comprehensive chromosomal screening overnight
Source: PLoS One. 2021 May 20;16(5):e0251971. doi: 10.1371/journal.pone.0251971 (PMC8136696; doi:10.1371/journal.pone.0251971)
Supplement: S1 Raw images — (PDF) [file pone.0251971.s001.pdf]

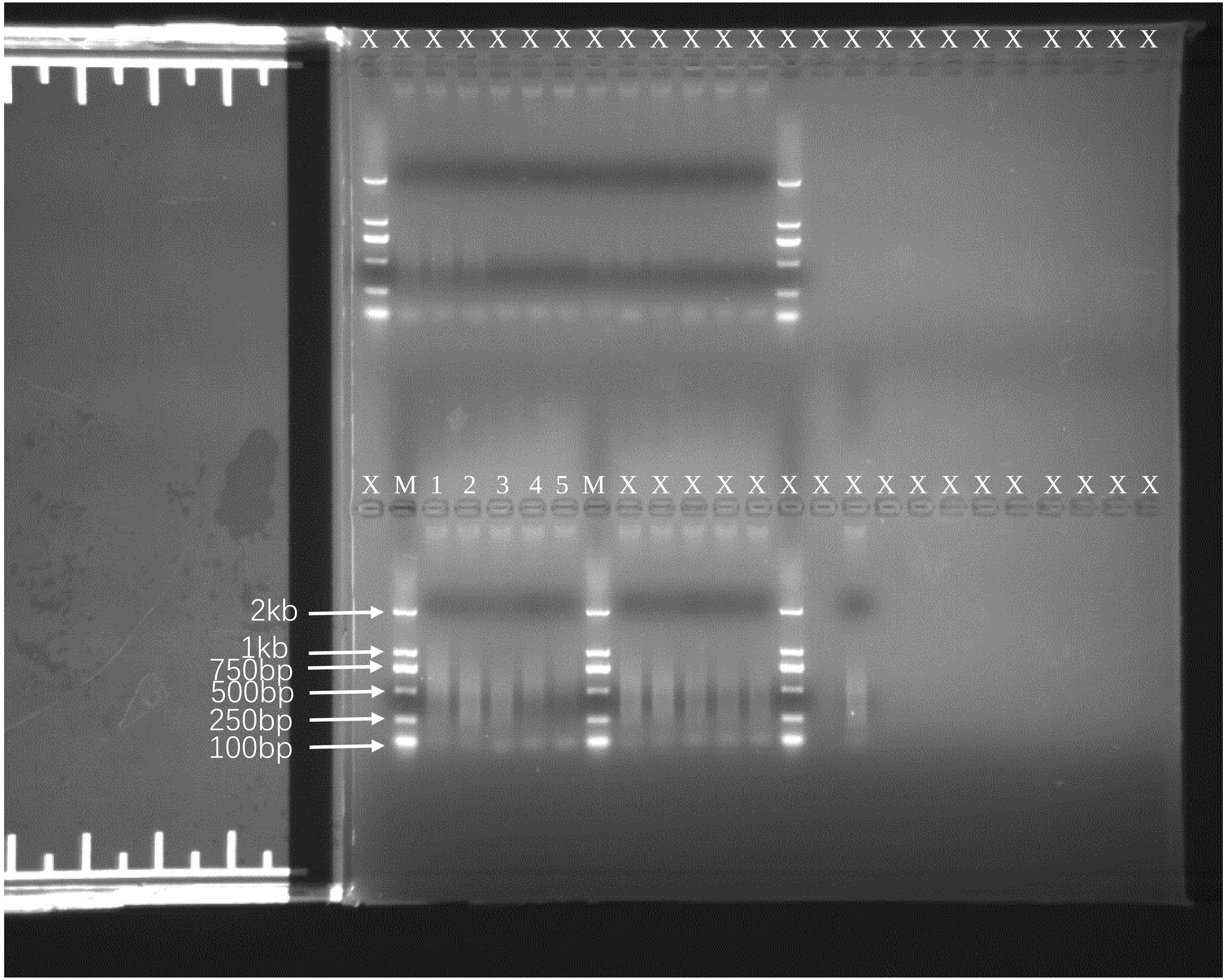


**Figure 1. The original image of figure 2.** WGA-NGS library construction result from variable pre-amplification primer designs. The amplification products are visualized on a 2% agarose gel as routine. M: DM2000 DNA Marker; Lane 1-5: the WGA products of design 1- 5. The lane marked with "X" means it's not included in the final figure.

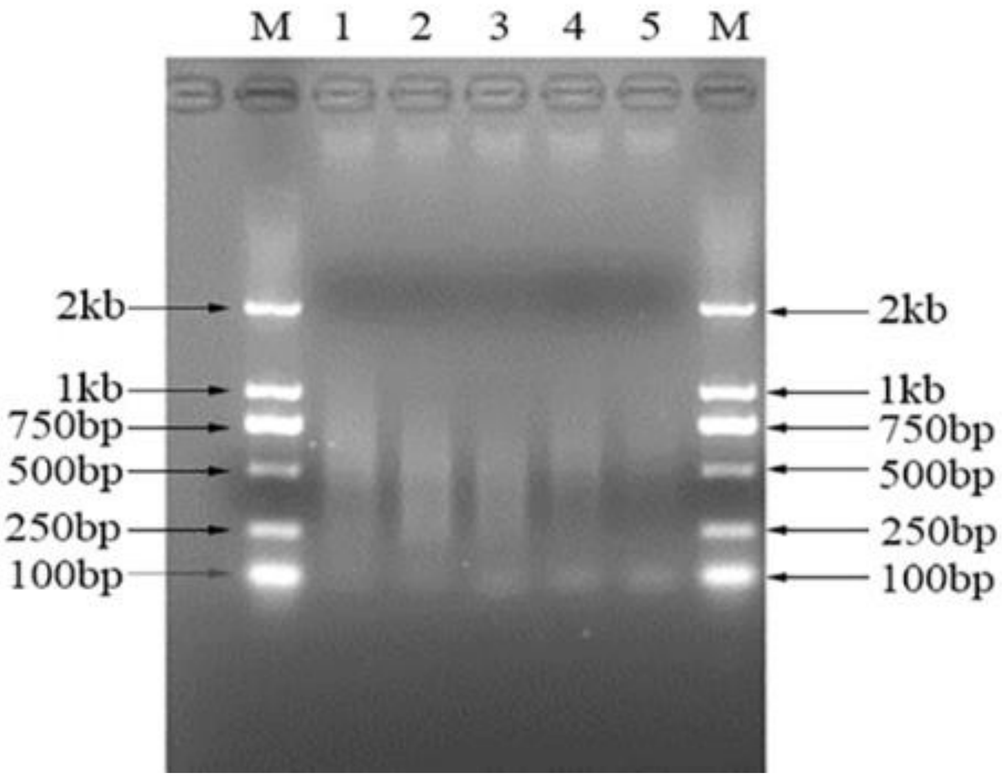


**Figure 2. WGA-NGS library construction result from variable pre-amplification primer designs.** Fifty picograns human genomic DNA was used as amplification template. The amplification products are visualized on a 2% agarose gel as routine. M: DM2000 DNA Marker; Lane 1-5: the WGA products of design 1- 5.
